# Human Neutrophil Alpha-Defensins Promote NETosis and Liver Injury in Alcohol-Related Liver Cirrhosis: Potential Therapeutic Agents

**DOI:** 10.3390/jcm13051237

**Published:** 2024-02-22

**Authors:** Anna Rycyk-Bojarzyńska, Beata Kasztelan-Szczerbińska, Halina Cichoż-Lach, Agata Surdacka, Jacek Roliński

**Affiliations:** 1 Department of Gastroenterology with Endoscopy Unit, Medical University of Lublin, 20-059 Lublin, Poland; beata.szczerbinska@umlub.pl (B.K.-S.); halina.lach@umlub.pl (H.C.-L.); 2 Department of Clinical Immunology, Medical University of Lublin, 20-059 Lublin, Poland; agata.surdacka@umlub.pl (A.S.); jacek.rolinski@umlub.pl (J.R.)

**Keywords:** alpha-defensins, human neutrophil peptides (HNPs), neutrophils, NETosis, neutrophil extracellular trap (NET), alcohol-related liver cirrhosis, alcohol-related liver disease

## Abstract

**Background**: Neutrophils are thought to play a pivotal role in the pathogenesis of many inflammatory diseases, such as hepatitis, liver cirrhosis, etc. Activated human neutrophils release human neutrophil peptides (HNP1-3) or alpha-defensins that are antimicrobial peptides in azurophil granules. Furthermore, HNP1-3 build a scaffold of neutrophil extracellular traps (NETs) and promote the process of programmed cell death called NETosis. Our study aimed to investigate the role of alpha-defensins in the pathogenesis of alcohol-related liver cirrhosis (ALC). **Methods:** The concentrations of alpha-defensins in the plasma of 62 patients with ALC and 24 healthy subjects were measured by ELISA. The patients with ALC were prospectively recruited based on the severity of liver dysfunction according to the Child-Pugh and Model of End-Stage Liver Disease-Natrium (MELD-Na) scores, modified Maddrey’s Discriminant Function (mDF), and the presence of ALC complications. **Results:** The concentrations of alpha-defensins in plasma were significantly higher in the ALC patients than in the controls. The plasma levels of HNP1-3 correlated with the MELD and mDF scores. ALC subgroups with MELD > 20 and mDF > 32 displayed significantly higher HNP1-3 concentrations. The plasma levels of HNP1-3 revealed a good predictive AUC for hepatic encephalopathy and ascites development (0.81 and 0.74, respectively) and for patient survival (0.87) in those over 40 years of age. **Conclusion:** These findings suggest that alpha-defensins play an important role in the assessment of ALC.

## 1. Introduction

Globally, alcohol is the main cause of liver cirrhosis (in Europe, North America, and Latin America), accounting for 60% of the total number of cirrhosis cases [1]. Alcohol-related liver disease (ALD), which is considered to be the most common indication for liver transplantation (LT), leads to liver cirrhosis [2]. The ALD spectrum consists of alcohol-related steatosis, steatohepatitis, fibrosis, acute liver failure, chronic liver failure, and hepatocellular cancer (HCC) in cirrhosis [2]. The risk of HCC among patients with alcohol-related liver cirrhosis (ALC) is about 1% per year [3]. The current state of knowledge regarding the molecular pathomechanism of ALC remains unclear and requires the investigation of the most innovative therapeutic options [2]. Nowadays, the only effective treatment option for ALC is liver transplantation. Current evidence suggests that neutrophils play a crucial role in the development of ALC. Moreover, NETosis, which is a form of programmed cell death, is a consequence of neutrophil extracellular trap (NET) formation and, subsequently, NET activation. Next, NETosis as one of the forms of programmed death in inflammatory diseases is the main subject related to identifying treatments for ALC. According to many studies, NETs may contribute to the inflammatory response of the host [4]. NETs are extracellular web-like structures of DNA-histone complexes with neutrophil granule proteins such as defensins [5]. In 1985, human neutrophil defensins (human neutrophil peptides 1-3, HNP1-3) were discovered by Lehrer [6]. They are mainly synthesized by neutrophils, epithelial cells, and Paneth cells [6]. To date, six human defensins have been detected [7]. For more than two last decades, they have been investigated in viral, bacterial, and fungal infections [7,8]. Mammalian defensins are classified as alpha- and beta-defensins [9]. Human alpha-defensins are located on chromosome 8 [6]. Structurally, they are a family of small peptides (less than 10 kDa) [10]. Alpha-defensins are first-line defense peptides of the innate immunity [8,11]. They are a part of the antimicrobial peptide (AMP) family and display antimicrobial activity [12]. A two-step cell injury process has been suggested by researchers [9]. It begins with permeabilization of the cell membrane, then DNA damage occurs [9]. Overall, recent findings have detected defensins’ inflammatory response in many diseases such as HBV and HCV infections, tumorigenesis, diabetes mellitus, SARS-CoV-2 infections, atopic dermatitis, etc. [9,12,13,14,15,16]. However, there are no data regarding the role of defensins in alcohol-related liver cirrhosis. Our objective was to examine the alpha-defensin serum concentrations in patients with alcohol-related liver cirrhosis.

## 2. Materials and Methods

### 2.1. Patients

Our study assessed 86 consecutive patients, namely, 62 patients with confirmed ALC and 24 matched healthy controls, who were admitted to the Department of Gastroenterology with Endoscopy Unit in Lublin. The patients were prospectively recruited to the study over 2 years. In total, 51 males and 11 females with an average age of 49 (59) years served as the study group. The control group consisted of 24 age- and sex-matched healthy volunteers, namely, 15 males and 9 males. According to the WHO, the healthy volunteers without alcohol abuse who served as controls consumed no more than 10 g of ethanol daily [17]. In the current study, the same patient recruitment protocol presented in our previous studies was used [18,19,20]. The inclusion criteria were as follows: written, informed consent of the patient to participate in the study; age >18 years; positive screening results according to the Alcohol Use Disorder Identification Test-Consumption (AUDIT-C) questionnaire; and an interview confirming alcohol abuse. We excluded patients with celiac disease, Wilson’s disease, alpha-1-antitrypsin deficiency, viral hepatitis, and autoimmune liver disease; those with any severe comorbidities (malignancy, respiratory failure, or cardiovascular disease); and those with blood transfusion, immunotherapy, or steroid treatment in the last 6 months. The studied patients were grouped based on the following:

(1) sex;
(2) the severity of liver failure classified by the Child-Turcotte-Pugh (CTP) score, Model for End-stage Liver Disease-Sodium (MELD-Na) score, and modified Maddrey Discriminant Function (mDF) score;
(3) ALC decompensation symptoms, such as ascites, hepatic encephalopathy, and esophageal varices.

The severity of liver failure was established using the above mentioned scores (the CTP, MELD-Na, and mDF scores) using internet available calculators. The diagnosis of ALC was based on the guidelines upon documentation of regular alcohol consumption confirmed by the patients or their family members [21]. According to the Helsinki Declaration guidelines, all patients provided informed written consent.

Special tools were used to identify alcohol use disorder (AUD) among ALC patients. In our study, scores for the AUDIT-C, which is a shorter form of the commonly used Alcohol Use Disorder Identification Test (AUDIT), were obtained for each patient to quantify lifetime drinking history. According to the AUDIT-C, 100% of patients in the study group received at least 3 points, which was a positive test result [22,23,24]. The AUDIT-C results were negative for all of the controls. The diagnosis of alcohol-related liver disease was based on symptoms, physical examination, laboratory abnormalities, and imaging tests in accordance with the European Association for the Study of the Liver (EASL) Clinical Practice Guidelines [1]. Imaging techniques showing liver cirrhosis included abdominal ultrasonography or computed tomography (CT) scans.

Subsequently, ALC patients included in the study underwent upper gastrointestinal tract endoscopy. In the study group, 45% of patients were confirmed to have esophageal varices. The presence of portal hypertension was detected in Doppler mode abdominal ultrasonography. It was observed in 58% of the ALC patients. According to the guidelines, performing liver biopsy was not necessary to establish the diagnosis [21]. Eighteen patients from the study group who presented symptoms of hepatic encephalopathy (HE) were evaluated according to the Clinical Hepatic Encephalopathy Staging Scale (CHESS) [25,26,27]. The patients with ascites underwent diagnostic paracentesis to exclude spontaneous bacterial peritonitis (SBP). Next, four patients were diagnosed with SBP and received antibiotic treatment. Large-volume paracentesis was necessary in 87% of ALC patients. According to the guidelines, patients after 5-L paracentesis were administered albumin infusion [21].

The individuals were followed for 90 days. All studied patients were discharged from hospital when liver function began to improve. Subsequent follow-up visits were planned for every 2 weeks of the 90-day observation period in the outpatient clinic or in the hospital department if necessary. Three patients died during the 90-day observation period.

This study was approved by the Local Ethics Committee of the Medical University of Lublin (No. KE-0254/94/2019).

### 2.2. Procedures

Venous blood samples were obtained from the studied and control patients after an overnight fast. All the tests were performed in the laboratory of the Department of Clinical Immunology at the Medical University of Lublin. The study was performed as recommended by the producer. In total, 15 milliliters of peripheral blood was obtained from ALC patients and control group subjects with a sterile S-Monovette (SAR-STEDT AG & Co., D-51588 Numbrecht, Germany). Next, the samples were incubated for 20 min in a dark dry room at room temperature. Plasma concentrations of HNP1-3 were quantified with an HNP1-3 enzyme-linked immunosorbent assay (ELISA, Hycult Biotech, Uden, The Netherlands) kit.

### 2.3. Statistical Analyses

Statistical analyses of the results were conducted with the use of the Statistica 10 software package (StatSoft, Cracow, Poland). Deviation from normality was evaluated by the Kolmogorov–Smirnov test. The Mann–Whitney U test was used for between-group comparisons, while Spearman’s correlation test was used to verify the correlations between the parameters of liver function and HNP1-3 concentrations. The categorical variables were described using either Fisher’s exact test or the χ^2^ test. Next, the Kruskal–Wallis test was used to check the differences in HNP1-3 levels in the patients with different severity of liver dysfunction according to the CTP score. The receiver operating characteristic (ROC) curves and areas under the curve (AUCs) were checked in order to assess the sensitivity and specificity of measured variables for predicting the degree of liver failure. The method of DeLong was used for the calculation of the standard error of the AUC. The Youden index was estimated for marking optimal points. A value of *p* < 0.05 was considered to be statistically significant.

## 3. Results

### 3.1. Comparison of Females and Males with Alcohol-Related Liver Cirrhosis

Routine laboratory tests were performed among the group of ALC patients. These tests assessed liver function parameters; levels of alanine aminotransferase (ALT), aspartate aminotransferase (AST), alkaline phosphatase (ALP), ɣ-glutamyl transpeptidase (GGT), total bilirubin, and albumin; prothrombin time (PT) with the international normalized ratio (INR); kidney function on the basis of the creatinine level; and markers of inflammation, namely, the C-reactive protein (CRP) level, white blood cell (WBC) count, neutrophil (NEU) count, lymphocyte (LYM) count, and neutrophil to lymphocyte ratio (NLR). Based on these parameters, the CTP, MELD-Na, and mDF scores were evaluated. Online available calculators were used. The results are presented in the next chapters. The patients with ALC were examined in order to detect the decompensation of chronic liver disease symptoms such as encephalopathy, esophageal varices, and ascites, and the mortality rate was calculated. The evaluation of the demographic and laboratory data in the ALC patients was performed as seen in Table 1.

A comparison of the levels of the laboratory data, the CTP, MELD-Na, and mDF scores, and the presence of symptoms of liver decompensation showed no differences between females and males with ALC.

### 3.2. Comparison of Serum HNP1-3 Concentrations in the ALC Patients and Individuals in the Control Group

Subsequently, serum HNP1-3 concentrations between females and males with ALC and between ALC patients and controls were evaluated. The results are presented in Table 2.

Our analysis did not reveal significant differences in serum HNP1-3 concentrations in ALC subgroups according to sex (*p* < 0.25). However, it showed statistically significant higher serum HNP1-3 concentrations (*p* < 0.0009) in the ALC group compared to the controls.

Next, serum HNP1-3 concentrations in ALC males and females were compared to males and females from the control group (Table 3).

The results showed no significant differences in serum HNP1-3 concentrations in ALC males and in healthy males (*p* < 0.044). However, the results showed significantly higher serum HNP1-3 concentrations (*p* < 0.003) in ALC females compared to control females.

### 3.3. Comparison of Serum HNP1-3 Concentrations in Patients with ALC Based on Age

Serum HNP1-3 concentrations in ALC patients were measured based on age > 40 years vs. <40 years (Table 4).

The results showed no differences in serum HNP1-3 concentrations (*p* < 0.66) in patients with ALC based on age.

### 3.4. Analysis of Correlations of Serum HNP1-3 Concentrations with Child-Turcotte-Pugh, MELD-Na, and mDF Scores

#### 3.4.1. Analysis of Correlations of Serum HNP1-3 Concentrations with Child-Turcotte-Pugh Scores

Differences in serum HNP1-3 concentrations in patients with ALC were evaluated based on the degree of liver dysfunction classified according to the CTP score (class A—17 patients, class B—25 patients, and class C—20 patients). The results are shown in Table 5.

There were no statistically significant differences in serum HNP1-3 concentrations in patients with ALC and different CTP scale scores (*p* < 0.30).

#### 3.4.2. Analysis of Correlations of Serum HNP1-3 Concentrations with MELD-Na Scores

Differences in serum HNP1-3 concentrations in patients with ALC were evaluated based on the degree of liver dysfunction classified according to the MELD-Na score (>20 points or ≤20 points). The results are presented in Table 6.

**Figure 1:**
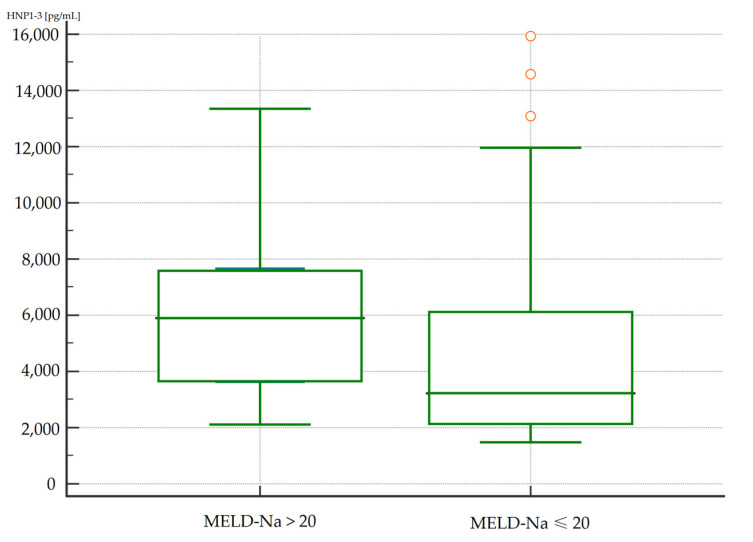
Comparison of serum HNP1-3 concentrations in patients with ALC with different MELD-Na scores. Legend: MELD-Na—Model for End-Stage Liver Disease-Sodium score; HNP1-3—human neutrophil peptides 1-3.

**Figure 2:**
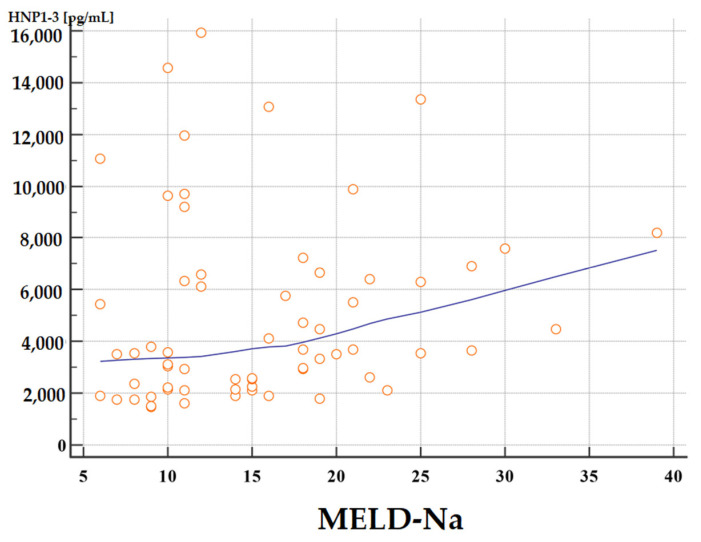
Correlation between serum HNP1-3 concentrations in patients with ALC with different MELD-Na scores. Legend: MELD-Na—Model for End-Stage Liver Disease-Sodium score; HNP1-3—human neutrophil peptides 1-3.

The concentrations of HNP1-3 were statistically significantly increased in patients with ALC who had a MELD-Na score of more than 20 points (*p* < 0.02) compared to patients with a MELD-Na score of ≤20 points. Figure 1 and Figure 2 present the results of comparison of serum HNP1-3 concentrations in ALC patients based on MELD-Na scores (Mann–Whitney test).

#### 3.4.3. Analysis of Correlations of Serum HNP1-3 Concentrations with mDF Scores

**Figure 3:**
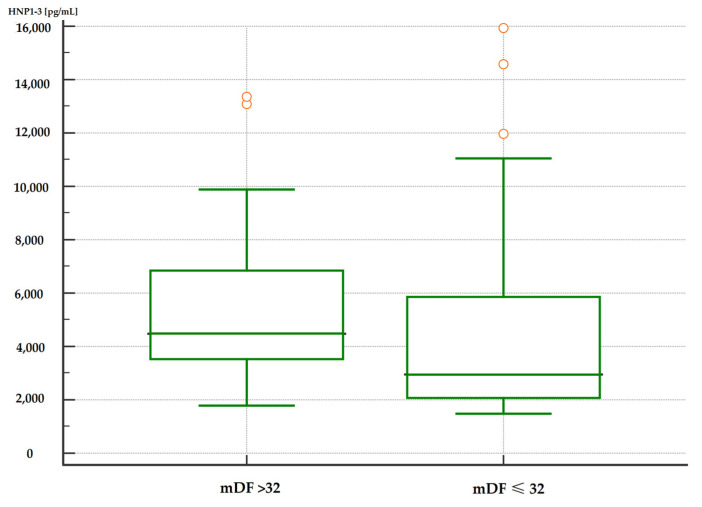
Comparison of serum HNP1-3 concentrations in patients with ALC with severe alcohol hepatitis based on mDF scores (*p* < 0.01). Legend: mDF—modified Maddrey Discriminant Function score; HNP1-3—human neutrophil peptides.

**Figure 4:**
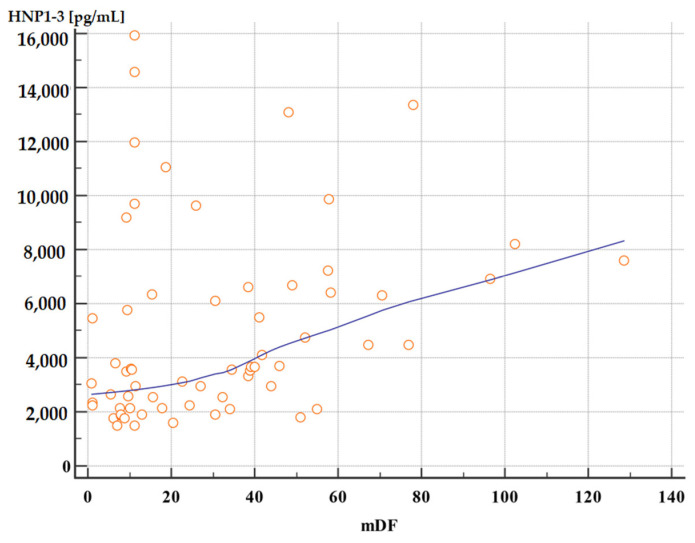
Correlation between serum HNP1-3 concentrations in patients with ALC with different mDF scores (*p* < 0.01, rho 0.40). Legend: mDF—modified Maddrey Discriminant Function score; HNP1-3—human neutrophil peptides.

Moreover, patients with severe alcoholic hepatitis with an mDF score of >32 points also had significantly higher serum HNP1-3 concentrations compared to patients with an mDF score of ≤32 (Table 7, Figure 3 and Figure 4).

### 3.5. Comparison of Serum HNP1-3 Concentrations in Patients with ALC Based on End-Stage Liver Disease Complications

Among different features of chronic liver disease decompensation, the highest predictive value AUCs for serum HNP1-3 concentrations were detected for the development of hepatic encephalopathy (0.81), ascites (0.74), and mortality at >40 years (0.87) in ALC patients. The highest sensitivity and specificity were found in the analysis of predictive value for serum HNP1-3 for death in ALC patients above 40 years of age.

#### 3.5.1. Comparison of Serum HNP1-3 Concentrations in Patients with ALC Based on the Development of Hepatic Encephalopathy

**Figure 5:**
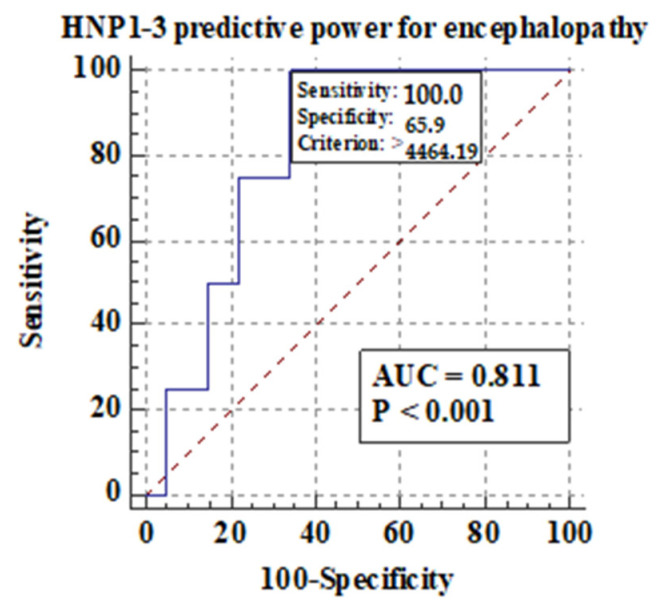
Predictive power of serum HNP1-3 concentrations for the development of hepatic encephalopathy in ALC patients.

Figure 5 shows the predictive power of serum HNP1-3 concentrations for the development of hepatic encephalopathy.

Figure 5 shows that there is a correlation between the development of hepatic encephalopathy and higher serum HNP1-3 concentrations in ALC patients with a sensitivity of 100% (*p* < 0.001).

#### 3.5.2. Comparison of Serum HNP1-3 Concentrations in Patients with ALC Based on the Development of Ascites and Esophageal Varices

**Figure 6:**
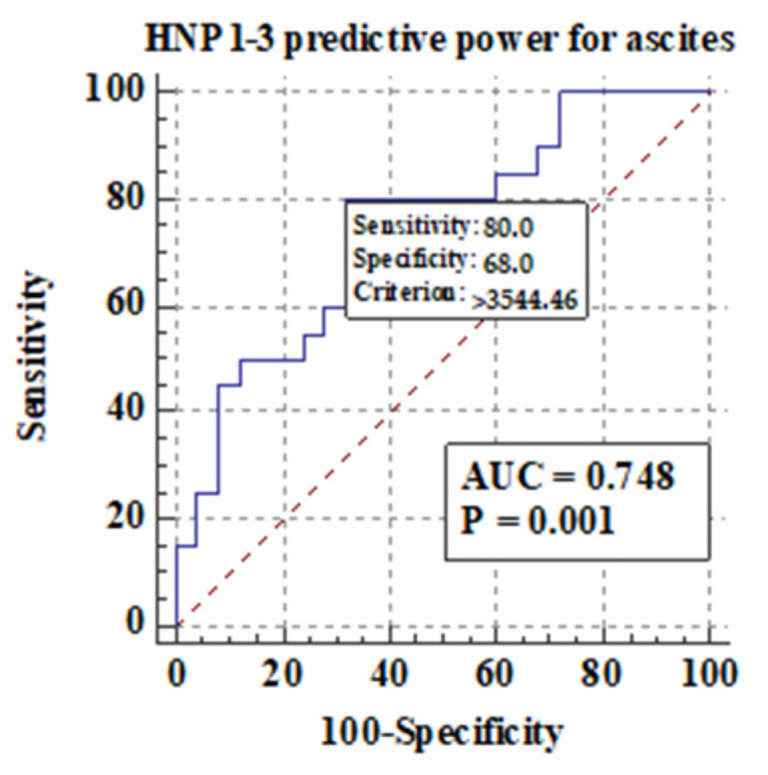
Predictive power of serum HNP1-3 concentrations for the development of ascites in ALC patients.

Figure 6 shows the predictive power of serum HNP1-3 concentrations for the development of ascites in ALC patients.

Figure 6 shows that there is a correlation between the development of ascites and higher serum HNP1-3 concentrations in ALC patients (*p* < 0.001). Next, in our study, serum HNP1-3 concentrations were compared according to subgroups based on the presence of esophageal varices. We concluded that there is no correlation between the presence of esophageal varices and serum HNP1-3 concentrations in ALC patients (*p* < 0.58).

#### 3.5.3. Comparison of Serum HNP1-3 Concentrations in Patients with ALC Based on Non-Survival

Patients with ALC were evaluated for 90 days. During this time, three ALC patients died, while 59 individuals survived. We compared serum HNP1-3 concentrations among these two subgroups of patients as shown in Table 8.

There were no significant differences in serum HNP1-3 concentrations in patients with ALC who died and in those who survived (*p* < 0.54) during a 90-day observation period.

**Figure 7:**
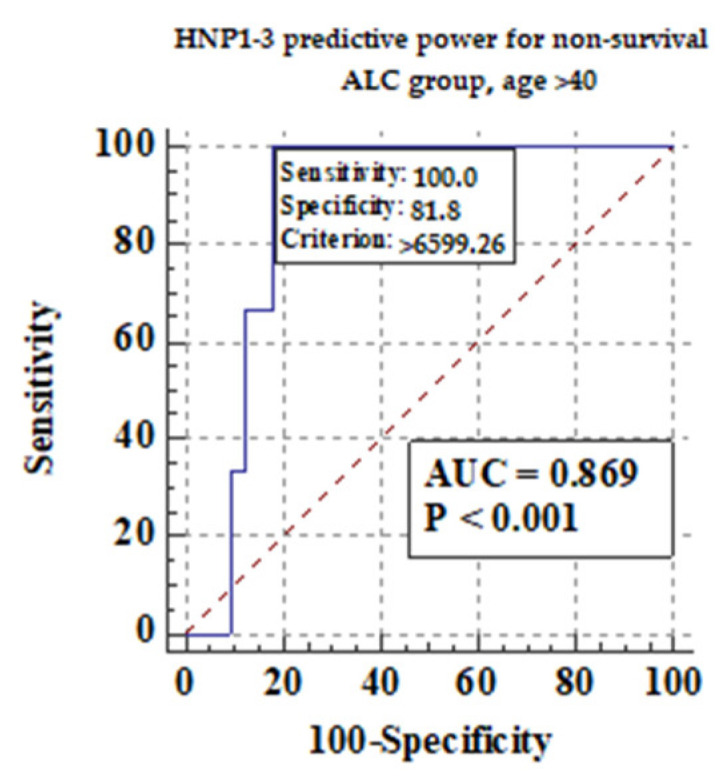
The predictive power of serum HNP1-3 concentrations for non-survival in ALC patients > 40 years.

We also measured if age could be an important factor for survival in ALC patients. We divided ALC patients into two subgroups: older than 40 years of age, and no more than 40 years of age. Figure 7 shows the predictive power of serum HNP1-3 concentrations for non-survival in ALC patients > 40 years.

Figure 7 shows that there is a correlation between non-survival and higher serum HNP1-3 concentrations in ALC patients (*p* < 0.001).

### 3.6. Analysis of Correlation of Serum HNP1-3 Concentrations and Markers of Inflammation in Patients with ALC

We checked whether there was any relationship between serum HNP1-3 concentrations and markers of inflammation (CRP, WBC, neutrophils, and NLR) in ALC patients. The results are presented in Table 9.

No positive correlations were found between HNP1-3 and CRP, WBC, NEU, and NLR levels.

## 4. Discussion

Alcohol-related liver disease has been reported as a very common disease worldwide with the highest rates confirmed in western Europe [28]. According to previous studies, a 1-year mortality rate is estimated as 25–30% among ALD patients [29]. Furthermore, alcohol-related liver disease is responsible for almost 60% of cirrhosis cases in the USA [30]. According to a British study, the number of patient admissions to UK hospitals with the primary diagnosis of liver disease rose by 22% in 2022 and almost doubled in the past ten years [31].

In patients with alcohol-related liver cirrhosis, neutrophils are activated. However, their ability to respond to pathogens is impaired. Neutrophils’ antimicrobial activity is possible due to neutrophil extracellular traps, phagocytosis, or production of reactive oxygen species (ROS) [32]. NET formation is activated through cytokines and chemokines [33]. Significantly higher expression of AMPs such as HNP1-3, LL-37, HBD-1, and HBD-2 is observed in many inflammatory processes [34]. Antimicrobial peptides can be distinguished into cathelicidins and defensins.

Among defensins, HNP1-3 belong to the alpha-defensin peptide family, while HBD-1 and HBD-2 belong to the beta-defensin peptide family [34]. Some mechanisms have been described to be involved in the expression of alpha-defensins, such as mitogen-activated protein kinases (MAPKs) or nuclear factor kappa B (NF-κB) [35]. MAPKs play a crucial role in the production of cytokines such as TNF-alpha and IL-2 [36]. NF-κB signaling is triggered by the stimulator of interferon genes (STING) [37]. The inactivation of NF-κB signaling is possible through the peroxisome proliferator-activated receptor (PPAR) family [38]. Alpha-defensins induce the production of IFN-alpha, IFN-beta, and IL-6 and enhance TLR9 activation through the NF-κB pathway [39].

Several studies have revealed that PPARα ligands may exhibit anti-steatotic, anti-inflammatory, and anti-fibrotic effects [40]. The deregulation of PPAR pathways leads to the development of non-alcoholic fatty liver disease (NAFLD), ALD, hepatitis virus-injury, and HCC [41,42]. The chronic expression of alpha-defensins induces increased systemic lipolysis and decreased hepatic fat accumulation [43]. In particular, the defensins neutralize bacterial toxins, disrupt the membrane of the bacterial cell wall, and inhibit the synthesis of the bacterial cell wall leading to killing bacteria or preventing their growth [7]. Their role is crucial in tumorigenesis as well. While beta-defensins play a suppressive or a proliferative role in tumor development, alpha-defensins are suggested to be produced by tumors themselves. Furthermore, HNP1-3 lead to tumor progression and invasiveness [7].

Our study investigated whether alpha-defensin concentrations are associated with sex, age, CTP, MELD-Na, and mDF scores, ALC decompensation, and inflammatory markers. As a result, a significant correlation between HNP1-3 serum concentrations and ALC was found. The concentrations of HNP1-3 were evaluated in patients with ALC compared to healthy patients. Interestingly, females with ALC had elevated levels of HNP1-3 compared to healthy females. The concentrations of HNP1-3 were statistically significantly increased in patients with ALC with a MELD-Na score of >20 points (*p* < 0.02) compared to patients with a MELD-Na score of ≤20 points. Moreover, our findings are in line with previous human data, suggesting increased expression of defensins in chronic inflammatory diseases, such as cystic fibrosis, bronchiolitis, and psoriasis [34].

Similarly, a significant correlation between HNP1-3 serum concentrations and alcoholic hepatitis was found, which is a very promising discovery because of the lack of possible therapeutic options in the treatment of alcoholic hepatitis. Several clinical trials are under investigation at present. Nonetheless, liver transplantation and alcohol abstinence are the keys to quick recovery [44]. In our study, the highest predictive AUC values for HNP1-3 concentrations were detected for the development of hepatic encephalopathy (0.81), ascites (0.74), and mortality >40 years (0.87) in ALC patients. The presence of hepatic encephalopathy leads to acute-on-chronic liver failure (ACLF) leading to a high rate of short-term mortality [45]. Another study, in which the participants were patients with decompensation of liver cirrhosis plasma HNP1-3, showed better predictive accuracy for 90-day mortality (area under the receiver operating characteristic (AUROC) curve of 0.885)46 [46]. In the study by Abu Fanne et al., high HNP1-3 levels in patients with coronary artery disease negatively affected mortality (19.54%) and recurrent revascularization (8.05%), while high HNP1-3 levels were positively associated with myonecrosis and the severity of the disease [47]. The study by Abu Fanne et al. shows the elevation of alpha-defensin concentrations in fibrotic liver areas [11]. In another study, mice after sepsis onset given a high dose of HNP-1 showed more severe liver injury and increased mortality but no lung injury, which shows the liver predisposition of HNP1 [48]. More recent work, however, has shed light on the antimicrobial activity of defensins. AMPs induced by nutrients could be eligible alternatives to antibiotic therapy [35]. In our study, systemic concentrations of HNP1, HNP2, and HNP3, were significantly increased in patients with ALC in comparison with healthy individuals.

## 5. Conclusions

HNP1-3 levels correlated with MELD-Na scores and mDF scores. ALC subgroups with MELD > 20 and mDF > 32 presented with significantly higher HNP1-3 concentrations. Furthermore, HNP1-3 levels revealed a good predictive AUC for hepatic encephalopathy and ascites development (0.81 and 0.74, respectively) and for the survival (0.87) of patients over 40 years of age. Overall, the results reveal that the systemic blood levels of NET components—HNP1-3—are elevated in ALC patients and support the value of alpha-defensins as biomarkers in cirrhosis assessment. Therefore, HNP1-3 may be potentially applied as predictors of survival in patients with ALC.

Our study has some limitations. Firstly, this was an observational study. Therefore, we are currently unable to generate causal conclusions. Secondly, 77% of the study population were males, which limited the study generalizability to both sexes. However, our study included all patients admitted to the hospital with ALC—most patients with ALC are statistically males compared to females.

## Figures and Tables

**Table 1:** Demographic and laboratory data in the ALC patients assigned by sex.

Variable	Males ALC *n* = 51		Females ALC *n* = 11		*p*
	Median	5–95 Percentile	Median	5–95 Percentile	
Age, years ^a^	47.00	33.00–64.00	56.00	26.00–61.00	0.28
ALT, U/L ^a^	42.00	17.50–228.25	38.00	22.00–480.00	0.93
AST, U/L ^a^	101.00	34.40–360.40	112.00	43.00–550.00	0.74
ALP, U/L ^a^	153.00	56.50–405.15	121.00	73.00–411.00	0.76
GGT, U/L ^a^	378.00	43.20–2558.20	444.00	155.00–2193.00	0.16
Bilirubin, mg/dL ^a^	2.90	0.60–16.57	1.70	0.60–34.10	0.82
Albumin, g/dL ^a^	3.08	2.00–4.13	2.94	2.43–4.62	0.73
INR ^a^	1.29	0.93–2.23	1.33	0.60–2.54	0.87
Creatinine, mg/dL ^a^	0.80	0.42–1.57	0.60	0.40–2.90	0.14
CRP, mg/L ^a^	20.23	1.69–144.82	23.90	0.53–109.70	0.74
WBC, ×10^9^/L ^a^	7.00	3.24–15.94	5.79	4.07–13.55	0.94
NEU, ×10^9^/L ^a^	4.52	1.51–13.85	3.57	2.74–12.52	0.86
LYM, ×10^9^/L ^a^	1.06	0.44–2.37	0.92	0.46–1.55	0.39
NLR ^a^	3.98	1.47–14.81	4.03	2.06–27.22	0.68
CTP, points ^a^	8.00	5.00–13.00	8.00	5.00–13.00	0.78
MELD-Na, points ^a^	15.00	6.25–25.00	11.00	7.00–39.00	0.89
mDF, points ^a^	24.40	2.21–75.39	15.40	1.06–102.44	0.88
Ascites, % of patients ^b^	26 (50.98)		5 (45.45)		0.65
Encephalopathy, % of patients ^b^	16 (31.37)		2 (18.18)		0.67
Esophageal varices, % of patients ^b^	24 (47.05)		4 (36.36)		1.00
Non-survival, % of patients ^b^	2 (3.92)		1 (9.09)		0.33

ALC—alcohol-related liver cirrhosis; ALT—alanine aminotransferase; AST—aspartate aminotransferase; CRP—C-reactive protein; CTP—Child-Turcotte-Pugh score; GGT—ɣ-glutamyl transpeptidase; INR—international normalized ratio; LYM—lymphocytes; mDF—modified Maddrey Discriminant Function; MELD-Na—Model for End-Stage Liver Disease-Sodium score; NEU—neutrophils; NLR—NEU/LYM ratio; WBC—white blood cell count; ^a^ Mann–Whitney test; ^b^ Fisher test.

**Table 2:** Comparison of serum HNP1-3 concentrations in patients with ALC assigned by sex and between ALC patients and controls.

Variable, ng/mL	ALC Men *n* = 51		ALC Women *n* = 11		*p* ^a^	ALC Patients *n* = 62		Controls *n* = 24		*p* ^a^
	Median	5–95 Percentile	Median	5–95 Percentile		Median	5–95 Percentile	Median	5–95 Percentile	
HNP1-3	3.55	1.63–12.80	3.65	2.35–14.58	0.25	3.57	1.76–12.86	2.07	1.20–7.30	0.0009

ALC—alcohol-related liver cirrhosis; HNP1-3—human neutrophil peptides 1-3; ^a^—Mann–Whitney test.

**Table 3:** Comparison of serum HNP1-3 concentrations between female and male ALC patients and between female and male controls.

Variable, ng/mL	ALC Males *n* = 51		Controls Males *n* = 15		*p* ^a^	ALC Females *n* = 11		Controls Females *n* = 9		*p* ^a^
	Median	5–95 Percentile	Median	5–95 Percentile		Median	5–95 Percentile	Median	5–95 Percentile	
HNP1-3	3.55	1.63–12.80.	2.16	1.12–8.89	0.044	3.65	2.35–14.58	2.02	1.18–4.25	0.003

ALC—alcohol-related liver cirrhosis; HNP1-3—human neutrophil peptides 1-3; ^a^—Mann–Whitney test.

**Table 4:** Comparison of serum HNP1-3 concentrations between patients with ALC based on age.

Variable, ng/mL	Age > 40 Years *n* = 28		Age < 40 Years *n* = 32		*p* ^a^
	Median	5–95 Percentile	Median	5–95 Percentile	
HNP1-3	3.56	1.67–13.23	3.63	1.94–11.50	0.66

ALC—alcohol-related liver cirrhosis; HNP1-3—human neutrophil peptides 1-3; ^a^—Mann–Whitney test.

**Table 5:** Comparison of serum HNP1-3 concentrations between ALC patients based on Child-Turcotte-Pugh scores.

Variable (ng/mL)	ALC												*p * ^a^
	CTP Class A *n* = 17				CTP Class B *n* = 25				CTP Class C *n* = 20				
	Median	Minimum	25–75 Percentile	Maximum	Median	Minimum	25–75 Percentile	Maximum	Median	Minimum	25–75 Percentile	Maximum	
HNP1-3	3.09	1.48	1.91–5.44	11.96	3.52	1.50	2.32–6.46	15.94	4.49	1.60	3.03–6.99	13.35	0.30

ALC—alcohol-related liver cirrhosis; CTP—Child-Turcotte-Pugh score; HNP1-3—human neutrophil peptides 1-3; ^a^—Kruskal–Wallis test.

**Table 6:** Comparison of serum HNP1-3 concentrations between ALC patients based on MELD-Na scores.

Variable (ng/mL)	MELD-Na > 20 (Points) *n* = 13		MELD-Na ≤ 20 (Points) *n* = 49		*p* ^a^
	Median	5–95 Percentile	Median	5–95 Percentile	
HNP1-3	5.90	3.64–7.65	3.23	2.54–3.92	0.02

ALC—alcohol-related liver cirrhosis; MELD-Na—Model for End-Stage Liver Disease-Sodium score; HNP1-3—human neutrophil peptides 1-3; ^a^—Mann–Whitney test.

**Table 7:** Comparison of serum HNP1-3 concentrations between ALC patients based on mDF scores.

Variable (ng/mL)	mDF > 32 (Points) *n* = 26		mDF ≤ 32 (Points) *n* = 36		*p* ^a^
	Median	5–95 Percentile	Median	5–95 Percentile	
HNP1-3	4.49	3.65–6.60	2.94	2.23–3.59	0.01

ALC—alcohol-related liver cirrhosis; mDF—modified Maddrey Discriminant Function score; HNP1-3—human neutrophil peptides 1-3; ^a^—Mann–Whitney test.

**Table 8:** Comparison of serum HNP1-3 concentrations between ALC patients based on survival.

Variable (ng/mL)	Non-Survival *n* = 3		Survival *n* = 59		*p* ^a^
	Median	5–95 Percentile	Median	5–95 Percentile	
HNP1-3	8.21	2.41–9.71	3.55	1.76–13.08	0.54

ALC—alcohol-related liver cirrhosis; HNP1-3—human neutrophil peptides 1-3; ^a^—Mann–Whitney test.

**Table 9:** Analysis of correlations of serum HNP1-3 concentrations in ALC patients and markers of inflammation.

Markers of Inflammation	HNP1-3	
	Rho	*p* ^a^
CRP (mg/L)	0.14	0.41
WBC (×10^9^/L)	0.20	0.23
NEU (×10^9^/L)	0.17	0.28
NLR	0.26	0.11

ALC—alcohol-related liver cirrhosis; CRP—C-reactive protein; HNP1-3—human neutrophil peptides 1-3; NEU—neutrophils; NLR—neutrophil to lymphocyte ratio; rho—Spearman’s rank correlation coefficient; WBC—white blood cell count; ^a^—Spearman test.

## Data Availability

Data supporting reported results can be found by contacting the correspondence author.
